# Biomarkers of Development of Immunity and Allergic Diseases in Farming and Non-farming Lifestyle Infants: Design, Methods and 1 Year Outcomes in the “Zooming in to Old Order Mennonites” Birth Cohort Study

**DOI:** 10.3389/fped.2022.916184

**Published:** 2022-07-06

**Authors:** Kirsi M. Järvinen, Erin C. Davis, Erin Bevec, Courtney M. Jackson, Catherine Pizzarello, Elizabeth Catlin, Miranda Klein, Akhila Sunkara, Nichole Diaz, James Miller, Camille A. Martina, Juilee Thakar, Antti E. Seppo, R. John Looney, the Collaborative Working Group, Jeanne Lomas

**Affiliations:** Division of Allergy and Immunology, Center for Food Allergy, Department of Pediatrics, University of Rochester School of Medicine and Dentistry, Golisano Children’s Hospital, Rochester, NY, United States; Division of Allergy, Immunology, and Rheumatology, Department of Medicine, University of Rochester School of Medicine and Dentistry, Rochester, NY, United States; Allergy, Immunology, and Rheumatology, Rochester Regional Hospital, Rochester, NY, United States; ^1^Division of Allergy and Immunology, Center for Food Allergy, Department of Pediatrics, University of Rochester School of Medicine and Dentistry, Golisano Children’s Hospital, Rochester, NY, United States; ^2^Department of Microbiology & Immunology, University of Rochester School of Medicine and Dentistry, Rochester, NY, United States; ^3^Division of Allergy, Immunology, and Rheumatology, Department of Medicine, University of Rochester School of Medicine and Dentistry, Rochester, NY, United States; ^4^Department of Biostatistics and Computational Biology, University of Rochester School of Medicine and Dentistry, Rochester, NY, United States; ^5^Department of Public Health and Environmental Medicine, University of Rochester School of Medicine and Dentistry, Rochester, NY, United States

**Keywords:** infant immunity, farming lifestyle, human milk, microbiome, atopic dermatitis, food allergy, birth cohort

## Abstract

Traditional farming lifestyle has been shown to be protective against asthma and allergic diseases. The individual factors that appear to be associated with this “farm-life effect” include consumption of unpasteurized farm milk and exposure to farm animals and stables. However, the biomarkers of the protective immunity and those associated with early development of allergic diseases in infancy remain unclear. The “Zooming in to Old Order Mennonites (ZOOM)” study was designed to assess the differences in the lifestyle and the development of the microbiome, systemic and mucosal immunity between infants born to traditional farming lifestyle at low risk for allergic diseases and those born to urban/suburban atopic families with a high risk for allergic diseases in order to identify biomarkers of development of allergic diseases in infancy. 190 mothers and their infants born to Old Order Mennonite population protected from or in Rochester families at high risk for allergic diseases were recruited before birth from the Finger Lakes Region of New York State. Questionnaires and samples are collected from mothers during pregnancy and after delivery and from infants at birth and at 1–2 weeks, 6 weeks, 6, 12, 18, and 24 months, with 3-, 4-, and 5-year follow-up ongoing. Samples collected include maternal blood, stool, saliva, nasal and skin swabs and urine during pregnancy; breast milk postnatally; infant blood, stool, saliva, nasal and skin swabs. Signs and symptoms of allergic diseases are assessed at every visit and serum specific IgE is measured at 1 and 2 years of age. Allergic diseases are diagnosed by clinical history, exam, and sensitization by skin prick test and/or serum specific IgE. By the end of the first year of life, the prevalence of food allergy and atopic dermatitis were higher in ROC infants compared to the rates observed in OOM infants as was the number of infants sensitized to foods. These studies of immune system development in a population protected from and in those at risk for allergic diseases will provide critical new knowledge about the development of the mucosal and systemic immunity and lay the groundwork for future studies of prevention of allergic diseases.

## Introduction

Allergies and asthma have been growing global issues in industrialized nations/regions, rising in prevalence with diagnosis in early infancy/childhood. Asthma and allergies have increased exponentially over recent decades of industrialization and urbanization. Atopic diseases, including food allergy (FA), atopic dermatitis (AD), rhinoconjunctivitis and asthma, are the most common chronic medical conditions affecting children in the United States and are recognized as a global public health concern (1). Heredity is a strong determinant of an allergic constitution, but such rapid increases in disease prevalence are likely attributed to changes in environmental, dietary, and microbial exposures associated with a Western lifestyle (2). The “atopic march” starts shortly after birth with AD and sensitization to foods developing in the first year of life and allergic asthma and rhinoconjunctivitis developing as children reach school age. Despite major initiatives for prevention, no strategies have thus far succeeded in substantially decreasing morbidity.

Development of specific IgE to food allergens indicating sensitization is an important marker for the potential development of clinical FA, both of which are often preceded by AD suggesting a causal relationship (3). Further, the more severe and the earlier the onset of AD, the more likely is development of FA. Emerging evidence suggests that epicutaneous allergen sensitization occurs more readily through an impaired skin barrier, as seen in AD. This increases allergen permeability, leading to the production of proinflammatory cytokines, such as IL-25, IL-33, and thymic stromal lymphopoietin (TSLP) from the epithelial cells. These alarmins initiate an allergic inflammatory cascade, leading to allergic sensitization, food allergy and other atopic diseases. However, biomarkers of food allergy are lacking, despite the emerging strategies for prevention that call for need to identify those at risk.

While genetics is a contributor to allergy development, it is unlikely to explain the rapid increase in prevalence of allergic diseases (4). It is hypothesized that the rapid rise in allergy prevalence is, in part, due to the gradual loss of microbial exposures as nations/regions have transitioned into industrialization. Studies from Europe (5) (ALEX, GABRIELA, and PASTURE) and in North America among the religious agrarian communities, such as Amish, Old Order Mennonites (OOM) and Hutterites (6–8), suggest that living on farms is associated with a significant reduction in the rates of asthma and atopic diseases. Protection against allergy has been attributed to several factors of the farming lifestyle including consumption of unpasteurized farm milk and exposure to farm animals and their stables (5, 7) and enriched bacterial diversity in home dust (9). Regulatory T cells, B cells, and innate cells appear to be targets of farming lifestyle exposures (7, 10–12). Children who live on farms also have reduced numbers of medically attended respiratory illnesses in the first 2 years of life (13). Furthermore, alterations in the gut microbiome have been suggested to contribute to the development of atopic disease (14–19). These studies have focused on asthma at school-age. However, farm lifestyle studies on AD and FA that commonly precede development of respiratory allergies in a so-called “atopic march” are less studied. Recent data from the PASTURE birth cohort demonstrated that compared to non-farming infants, those exposed to a farming lifestyle harbored a more mature microbiome at 12 months characterized by increased abundance of SCFA producing bacteria, *Coprococcus* and *Roseburia* (19). Similarly, fecal butyrate and propionate concentrations and abundance of butyryl-CoA:acetate CoA-transferase (BCoAT), the gene encoding for the major enzyme in butyrogenesis, were lower among children who developed asthma. However, no single bacterial genus was associated with protection against asthma, suggesting that collective metabolic capacity of the bacteriome or species/strain dependent variation in SCFA production are important. Data from our studies demonstrate a divergent gut microbiome among OOM infants, in particular the enrichment of *Bifidobacterium longum* subspecies *infantis* (20). Presence of this subspecies has been suggested to induce an immunoregulatory/anti-inflammatory phenotype and be protective against allergy (21).

While there are several lines of evidence suggesting factors associated with the protective “farm-life effect,” the biomarkers associated with protection from allergic diseases in infancy remain unclear. Similarly, the biomarkers of development of allergic diseases in early life are scarce, with the exception of the specific IgE which indicates that sensitization has already occurred. Thus, we embarked on our “Zooming in to Old Order Mennonites (ZOOM)” study – a comprehensive longitudinal study to identify microbiome composition and biomarkers of the development of allergic diseases and those of protection against allergic diseases. Our two populations of interest are the OOM from the Finger Lakes region of New York who live a traditional agrarian lifestyle with low risk of allergic diseases and infants from the urban/suburban Rochester, New York (ROC) born to atopic families, who are at high risk for allergic diseases (8, 22). This report is a description of our ZOOM birth cohort study, including the premise, study design, biospecimen collection, initial demographic, lifestyle and exposure information, and allergic outcomes by 12 months of age. These studies of immune system development in a population “protected” from allergic diseases will provide critical new knowledge about the development of the mucosal and systemic immunity and lay the groundwork for future studies on prevention of allergic diseases.

## Methods and Analysis

### Study Populations and Recruitment

ZOOM is a prospective birth cohort study of infants at low- and high- risk for allergic diseases, which was enrolled between June 2017 and June 2020. The low-risk OOM are Groffdale Conference Mennonites of Swiss-German ancestry and reside predominately in Penn Yan, New York, 65 miles southeast of Rochester with a community size of about 5,000 people. Findings from our earlier studies assessing rate of allergic diseases and food introduction in this population using survey data (22) and our cross-sectional study has been published (20, 23). For the purposes of this longitudinal cohort study, pregnant mothers were recruited by a midwife or birth attendant during prenatal visits and by word-of-mouth prior to giving birth. High-risk ROC urban and suburban infants come from families with a first degree relative with allergic diseases diagnosed by physician or self-report. They were recruited among pregnant women who were seen or whose children were seen in the allergy practices in the greater Rochester area including our allergy practices at the University of Rochester Medical Center (URMC), or the Allergy, Asthma, Immunology and Rheumatology of Rochester private group and the Allergy Clinic at the Rochester Regional Hospital. These practices take care of the majority of the pediatric allergy patients in the area (estimated at least 2,000 families with food allergy per year). Additional recruitment also occurred among local pediatric and OBGYN practices and through social media.

We recruited mother-infant pairs prenatally and are collecting numerous samples and questionnaire information across the first 5 years of childhood, as well as assessing five clinical outcomes of: (1) food allergy (FA), (2) atopic dermatitis (AD), (3) allergic rhinitis (AR), (4) recurrent wheeze/asthma, and (5) allergic sensitization. We also collected samples and information from their mothers including stool, blood, and breast milk for microbial and immunological analyses and self-reported questionnaires as several aspects of maternal lifestyle and physiology could potentially influence infant risk for allergy development.

### Screening and Eligibility Criteria

**FIGURE 1:**
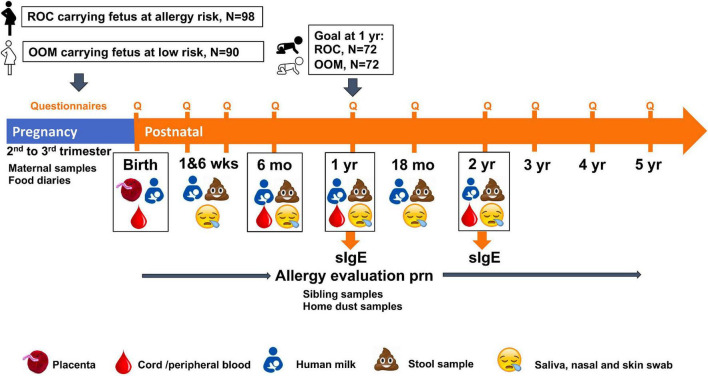
Overall study schematic. Pregnant Old Order Mennonite (OOM) and Rochester women carrying fetuses at risk for allergic disease were enrolled prenatally to a longitudinal birth cohort of infants to assess development of microbiome, systemic and mucosal immune system as well as allergic diseases. Visit and sample collection time points are shown. Specific IgE to select food and aeroallergens are measured at 1 and 2 years of age. Allergy evaluation is performed as indicated by any allergic symptoms throughout the follow up.

The study schematic is shown in Figure 1. Pregnant women were recruited during 2nd or 3rd trimester of gestation with the exception of the two infants recruited postnatally by 6 weeks of life. Women needed to be healthy and free of chronic infection, chronic inflammatory disease and known immunodeficiency. In the ROC cohort, they needed to be carrying a fetus with a first degree biologic relative with allergic disease (atopic eczema, food allergy, allergic rhinitis or asthma diagnoses physician-diagnosed or symptoms by self-report). In OOM community all families irrespective of the family history of allergic diseases were eligible. Eligibility criteria were reviewed during the first contact either by phone or in-person. To be eligible for enrollment into the study, infants needed to be born full term (>36 weeks of gestation), weigh >2,000 g at birth, and be generally healthy. The IRB protocol (RSRB52971) was approved by the Institutional Review Board of the University of Rochester Medical Center. Informed consent was provided by the mother prior to enrollment and on behalf of the infant right after birth.

### Study Visit Schedule

The schedule of bio sample collection during pregnancy and the first 2 years of life is shown in Table 1. Mothers had a baseline prenatal visit at enrollment, and visits at 1–2 weeks, 6 weeks, and 4–6 months postpartum. Infants had cord blood collected at birth and in-person visits at home or in the clinic at 1–2 weeks, 6 weeks, and 6, 12, and 18 months with 24-month follow-up ongoing. Permitted window of visits is ±1 week at 6 weeks and −1 month to +3 months for later visits. Follow up at 3, 4, and 5 years is by telephone or by answering survey alone with no in-person contact.

**TABLE 1:** Sample collection schedule.

	Prenatal	Birth	1–2 weeks	6 weeks	6 months	12 months	18 months	24 months	Currently planned assays
House dust*				x	x	x	x	x	
Maternal stool*	**x**			**x**					Microbiome (shotgun metagenomics)
Maternal blood*	**x**			**x**					Targeted metabolomics, Total and antigen specific antibody responses
Maternal saliva*	x			x					
Maternal nasal swabs	x								
Maternal skin swab*	x			x					
Maternal urine	x								
Placenta		x							
Cord blood		**x**							Immunophenotyping, specific antibody responses, BCR Rep-Seq
Breast milk		x	x	**x**	x				HMOs, fatty and organic acids, untargeted metabolomics
Child buccal swab				x					Host genetic analyses
Child stool			**x**	**x**	**x**	**x**	**x**	x	Microbiome (shotgun metagenomics)
				**x**	**x**	**x**			Targeted/untargeted metabolomics, IgA-Seq
Child blood					**x**	**x**	x	x	Immunophenotyping, total and antigen specific antibody responses, cytokines, BCR Rep-seq
Child saliva			**x**	**x**	**x**	**x**	**x**	**x**	Antigen specific antibody responses
Child nasal swabs				x	x	x	x	x	
Child skin swab			x	**x**	x	x	x	x	Microbiome (16S rRNA analysis)

Bolded “x” denotes the time points at which the outlined assays have been planned. *denotes samples that were only collected one time but may have been collected at different time points among subjects. HMO, human milk oligosaccharide. rRNA, ribosomal RNA. BCR, B cell receptor. Rep-Seq, repertoire sequencing.

### Questionnaires

In addition to collecting research samples, we also administer multi-page questionnaires at prenatal visits, 6 weeks, 4–6, 12, 18, and 24 months, and at 3, 4, and 5 years. The questionnaires are administered online, on paper, or through direct interview with one of the parents, and include questions about allergic symptoms such as vomiting, eczema, abdominal pain/colic, cough, wheeze and symptoms suggestive of food allergy with specific foods (skin rash, hives, difficulty breathing, wheezing/cough, itching/swelling of lips/mouth/throat, throat closing, abdominal pain/vomiting/diarrhea/bloody stools and anaphylaxis). We also queried physician-diagnosis of eczema, food allergies, allergic rhinitis/hay fever, and wheezing/whistling in the chest, asthma, or cough, runny, stuffy or itchy nose or watery eyes without cold. Where possible standardized questions were drawn from instruments such as the Household Environment and Lifestyle Survey (24) and Multidimensional Scale of Perceived Social Support (25). Questionnaire domains and administration timeline are shown in Supplementary Table 1. A 3-day food diary was collected from mothers at screening/prenatal and/or 6 weeks and are also collected from children at 12 and 24 months, and 3, 4, and 5 years. Subjects declining certain questionnaires will still be included.

### Assessment of Clinical Outcomes

In addition to allergy symptoms assessed in surveys (see above), the investigator and/or sub-investigators query signs and symptoms of rash or concern for food allergy/intolerance during study visits, by questionnaires and take photographs. Those with concerns are discussed and photos are reviewed with the study physician (KMJ), and follow is up done by a video call, phone call and/or in their home or the office. Any children in both cohorts who display symptoms of allergic disease, such as chronic rash, food reactions, wheeze/persistent cough and chronic rhinitis, are evaluated by the study physician, and allergen-specific IgE tests (ImmunoCAP) and skin prick tests are performed guided by these symptoms. Skin prick tests are performed using standard allergen extracts (Greer), allergen diluents as the negative control and histamine (10 mg/ml) as positive control. Occasionally subjects are referred to an allergist outside of our clinic, dermatologist or gastroenterologist by their own primary care physician (PCP), in which case PCP and/or specialist records are requested and diagnoses reviewed. In addition, serum specific IgE testing is performed at 1 and 2 years of age in every subject.

The diagnosis of AD is based on recurring or chronic red, pruritic rash in a distribution age-typical for atopic dermatitis. FA is diagnosed based on the NIAID sponsored Expert panel guidelines on diagnosis and management of FA (26). Accordingly, IgE-mediated FA is defined as a history of an immediate-onset reaction [hives, swelling of the lips/eyes/tongue, cough/wheezing/hoarse voice, vomiting within 1–2 h of ingestion of specific food and lasting no longer than 24 h and either (1) presence of specific IgE > 0.35 kU/L, or a wheal 3 mm or greater than that elicited by the saline control on skin prick testing or (2) a positive oral food challenge]. Among the non-IgE-mediated FA, allergic proctocolitis is diagnosed by medical history of visible specks or streaks of blood in stool in a generally healthy infant and resolution of symptoms when causative food is eliminated and recurrence following food challenge. Lack of systemic symptoms, vomiting, diarrhea and growth failure which differentiate it from other gastrointestinal FA disorders, food protein-induced enterocolitis syndrome (FPIES) and eosinophilic gastrointestinal disorders (EGID) diagnosed based on NIAID guidance (26). Food or aeroallergen sensitization is defined by a positive skin test or specific IgE. Allergic rhinitis/conjunctivitis (ARC) is defined by recurring or chronic rhinitis symptoms (discharge, congestion, sneezing, and itching) after specific allergen exposure or associated with positive aeroallergen specific IgE testing. Diagnosis of wheezing illness/asthma is a clinical one in children <6 years of age due to inability to assess pulmonary function. Recurrent wheeze is defined as parental report of at least 2 wheezing episodes (with whistling or wheezing sound in the chest). Wheezing illness is defined as (1) physician-diagnosed wheezing at an office visit, or (2) wheezing episode for which controller medication (beta-agonists and/or leukotriene inhibitors) was prescribed, or (3) a diagnosis of wheezing illness, bronchiolitis, reactive airway disease, or asthma. Asthma in this age is a clinical diagnosis and is defined as at least 2–3 episodes of wheezing in the setting of AD or more episodes without AD.

Here we report atopic diseases and sensitization by 12 months of age. The children who have been enrolled in the study may develop allergic diseases past the first 12 months of age. Subjects from both cohorts will continue to be followed by questionnaires and phone interviews as well as clinic visits when indicated based on allergic symptoms at 2, 3, 4, and 5 years for development of additional atopic outcomes including FA, AD, ARC and wheezing illness/asthma.

### Assessment of Specific IgE

At 12 and 24 months of age, presence of specific IgE is measured by ImmunoCAP (Thermo Fisher) from plasma after heparin blood was diluted 1:2 in phosphate buffered saline (PBS) to cow’s milk, hen’s egg white, peanut, cat, dog, and house dust mite. Results are expressed as kU/L, with 0.3 kU/L as the lowest level of detection after multiplication with 3 to account for dilution factor. We are collecting these data also from 24-month visit, but data are not yet complete.

### Study Endpoints and Outcomes

The primary endpoint is the incidence of physician-diagnosed FA at 1 and 2 years of age. The secondary endpoints are the incidence of physician-diagnosed AD at 1 and 2 years of age, incidence of physician-diagnosed ARC at 1 and 2 years, incidence of physician-diagnosed wheezing illnesses/asthma, incidence of sensitization to food and aeroallergen at 1 and 2 years of age. Exploratory endpoints are the following parameters between those with and without any atopic diseases or FA or AD: stool microbiome analysis (microbial composition), stool metabolic profiling, cord and peripheral blood biomarker profiling/immune cell immunophenotyping, and fecal calprotectin.

### Sample Size

Cohort size was powered to have clinical outcomes significantly different between OOM and ROC groups with clinical FA being of primary interest. The published data on the prevalence of food allergies in high-risk inner city United States population at 12 months suggests a rate of 5% of confirmed FA and on top of this twice the rate of possible FA (27). Rate of AD in this population was 30% (28). With known familial aggregation of food allergies and sibling having an increased rate of FA (OR 2.6), we estimated the rate of FA in our ROC very-high risk population (having a sibling with FA) to be 15%. We calculated that a sample of 72 in each cohort arm would provide 80% power to detect a statistically significant difference in incidence of FA as a primary outcome and a sample of 47 in each arm for AD as a secondary outcome. We planned to account for 15% dropout rate but after seeing a sizable prematurity rate in Rochester, expanded enrollment throughout the study to secure at least 72 in each arm at 12 months of age.

### Sample Collection and Processing

Sample collection utilizes pre-assembled collection kits that consist of barcoded collection vials and all necessary items required for sample collection. All plasticware and solutions that are included in sample collection kits are UV irradiated to eliminate contaminations of bacterial DNA.

#### Blood

Maternal blood was drawn from a vein in the arm in a quantity of no more than 50 mL by a trained phlebotomist or research coordinator. Adult blood was collected in 8 ml sodium heparin anticoagulated vacuum tubes. Discarded cord blood samples were collected after the placenta was delivered and separated from the baby. For cord blood, collection tubes supplemented with an additional 150 USP of sodium heparin were used to ensure clot-free sample preparation. Blood samples are drawn by venipuncture from infants/toddlers by a phlebotomist or research coordinator with experience in pediatric/infant blood draws using 2 ml sodium heparin vacuum tubes. No more than 3 ml per kg body weight of infant blood is drawn. Samples are collected at minimum 4 weeks post-vaccination to eliminate the impact of vaccinations on immune cells. Samples are diluted with two-fold volume of PBS and are processed with density centrifugation (Ficoll-Paque method) to separate and harvest plasma and peripheral blood mononuclear cells (PBMC). PBMCs are cryopreserved by programmed cooling (1°C/min) in 10% dimethyl sulfoxide in low endotoxin fetal calf serum and subsequently stored in liquid nitrogen. Initial sample volume as well as cell counts at the conclusion of PBMC prep are obtained and recorded. Any samples more than 24 h past collection or with visible signs of clotting at the onset or during processing are discarded. Samples are stored at −80°C or at liquid nitrogen as appropriate.

#### Stool Samples

Mothers were provided instructions and a stool collection kit including a stool hat for adult collection and diaper liners for the infants and toddlers. Once infant/toddler stool is passed, the sample is transferred to a sterile tube using a sterile, disposable spatula and immediately frozen in the home freezer and transported frozen to the laboratory by courier or study coordinator where it is then stored at −80°C.

#### Placenta

Placenta was transferred to the laboratory immediately after delivery, and three biopsies of approximately 0.2 ml by volume from both decidual (maternal) and chorionic villous (fetal) sides of the placenta were collected. Samples were transferred to conical tubes, preserved in RNAlater (Invitrogen), and frozen at −80°C.

#### Breast Milk

After not breastfeeding for at least 30 min, mothers were instructed to wash hands with soap and water, and clean breast with a castile soap towelette. Next, subjects collected 10–30 mL of milk using a manual breast pump with breast shield and transferred milk into 1–3 sterile 15 mL tubes. Samples were placed in the home freezer and stored at −80°C upon receipt in the laboratory.

#### Saliva

For infant/child saliva samples, parent or study coordinator holds one end of a cotton swab and gently encourages the child to suck on the swab. Swab is held in the mouth for 60–90 s until saturated and then placed in a sterile tube. Mothers were asked to gently spit 2 mL of saliva into a sterile container. Samples are transported to the laboratory and stored at −80°C upon receipt in the laboratory.

#### Nasal Samples

For infants/toddlers, study coordinator rubs the right nostril gently but with moderate pressure using a flocked swab for 5 s. Next, a nasal wash is performed using physiological saline to collect mucus. Nasal brush samples are also collected from infants/toddlers by rotating a cytology brush to cover all surfaces of the nostril for 5 s. Only a nasal swab was collected from mothers. Swabs are clipped into individual sterile tubes. Samples are transported back to laboratory and subsequently stored at −80°C.

#### Urine

After allowing a small amount of urine to be voided, mothers collected 1–2 ounces of urine using a sterile specimen container. Samples are placed in the home freezer and stored at −80°C upon receipt in the laboratory.

#### Skin Swabs

After dampening swab in sterile PBS, study coordinator rolls swab up and down and side to side on the subject’s inner forearm in a 2″ square. The swab is then clipped into 2 mL of PBS. Samples are transported to the laboratory and stored at −80°C.

#### Buccal Swab

Parents gently rubbed a Copan flocked swab up and down the inner side of the infant’s cheek with moderate pressure for 5 s while also slightly brushing the mucosa. Swabs were cut into a sterile tube and transferred to a −80°C upon receipt in the laboratory.

#### House Dust

We collected house dust from randomly selected families. A Dust stream collector (Indoor Biotechnologies) sample extractor was used to collect dust from home living room next to the couch by vacuuming for a minute. Settled house dust samples were collected via electrostatic dust collectors placed in living room bookcase for a recorded period. Exposure of adults to house dust will be assessed using personal dust samplers. Home dust will be collected once at any visit before the end of study.

### Statistical Analysis

For categorical variables, Fisher’s exact test was used to determine statistical significance between OOM and ROC groups. A Mann–Whitney *U* test was used for continuous variables, which all followed a non-normal distribution as assessed by a Shapiro–Wilk test. All analysis was conducted in Prism 9.0.

Survey data from the 12-month visit surveys were combined to reduce the collinearity among the variables. Variables with more than 80% missing data were excluded from the analysis. Multiple Correspondence Analysis (MCA) was conducted on this data using grouping of OOM vs. Rochester and allergic diseases vs. no allergic diseases outcomes (by physician diagnosis). The analyses were conducted using the FactoMineR package in R.

## Results

### Status of the Cohort and Follow-Up

**FIGURE 2:**
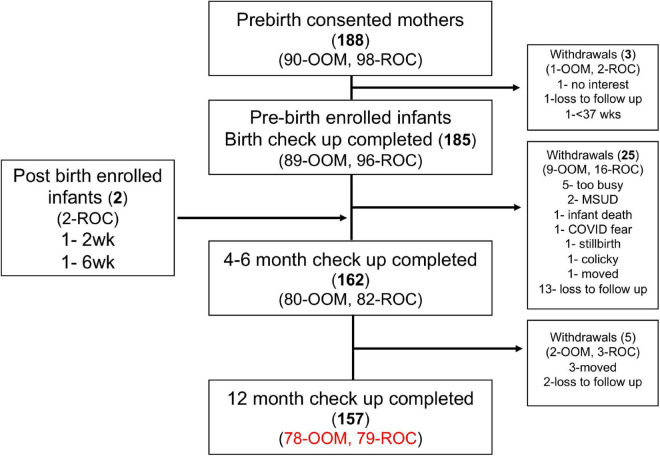
Old Order Mennonite (OOM) and Rochester (ROC) cohort recruitment through the first year of life. Follow up with the infants will continue at 18 and 24 months, as well at 3, 4, and 5 years of age. MSUD, maple syrup urine disease.

Altogether, 90 OOM and 98 ROC dyads were initially recruited (Figure 2). However, by the end of the 12-month follow-up, 78 OOM and 79 ROC dyads were still enrolled. Reasons for withdrawal from the study included lack of interest, loss to follow-up, preterm birth, family was too busy, infant diagnosed with Maple Syrup Urine Disease (MSUD), stillbirth, infant death, COVID fear, infant colic, family move. To date, all the infants have completed the 18-month follow-up visit; the oldest are 4.5 years old. An inventory of samples collected to-date can be found in Supplementary Table 2. Children were initially recruited for a follow-up until 24 months of age; at that point they are asked to enroll for annual telephone follow-up until the age of 6 years. During the annual contact, allergic symptoms, vaccinations, and other health outcomes are queried, and either medical records regarding visits for allergic symptoms are reviewed or children are referred to the study allergist for evaluation on a clinical basis.

### Family Characteristics and Exposures

Data from study questionnaires demonstrate that both OOM mothers and fathers were younger than ROC parents at baseline (Table 2). At 6 weeks postpartum, less OOM mothers reported influenza or Tdap vaccination in the previous year; fewer OOM mothers also reported infection requiring antibiotics since study enrollment, antibiotic use since delivery, and current medication use. Data on perinatal antibiotic use was not available. However, OOM mothers have low rates of cesarean sections and high rates of home births, which do not include Group B *Streptococcus* screening; therefore, their perinatal antibiotic use was presumably very low. Similar to results from our pilot study in these populations (20), OOM parents had significantly lower rates of asthma, seasonal allergies, and FA at enrollment. OOM mothers also had lower rates of eczema compared to ROC mothers; however, this was only a trend among fathers. Both OOM mothers and fathers had higher rates of exposure to various farm animals, and OOM mothers also had increased exposure to dogs and unpasteurized cow’s milk. Data on paternal exposures to household pets and unpasteurized cow’s milk was not available. In terms of home and lifestyle characteristics, OOM families had a greater number of older children. OOM were more likely to have a private water source and use pesticides inside and outside of their home (Table 3). However, fewer OOM used anti-bacterial cleaning agents. Significantly more OOM households utilized bicycles, walking, and horse and buggy as means of transportation. Lastly, OOM families regularly used dairy, egg, and peanuts in the home whereas ROC families had higher usage of soy and shellfish.

**TABLE 2:** Parent demographic characteristics.

Characteristic	*N* (%) OR MEAN (95% CI)		
	Rochester (*n* = 79)	OOM (*n* = 78)	*P*-value
Maternal age at enrollment (years)	31.8 (31.0, 32.7)	27.9 (26.8, 29.0)	**<0.0001**
n/a	0	1	
Paternal age at enrollment (years)	33.8 (32.3, 35.4)	28.4 (27.4, 29.4)	**<0.0001**
n/a	6	6	
Maternal pre-pregnancy BMI (kg/m^2^)	25.4 (24.1, 26.6)	25.8 (24.5, 27.1)	0.53
n/a	6	5	
Maternal race, Caucasian	71 (0.90)	78 (1)	**0.007**
Residence during pregnancy			**<0.0001**
City	51 (0.70)	0 (0)	
Village	13 (0.18)	0 (0)	
Farm	9 (0.12)	73 (1)	
n/a	6	5	
Maternal TDAP vaccination in past year^1^	56 (0.81)	3 (0.04)	**<0.0001**
n/a	10	1	
Maternal influenza vaccination in past year^1^	54 (0.78)	0 (0)	**<0.0001**
n/a	15	1	
Maternal antibiotic use since delivery^1^	17 (0.14)	10 (0.26)	0.09
n/a	13	6	
Maternal medication use (current)^1^	31 (0.48)	16 (0.21)	**0.001**
n/a	14	1	
Maternal infection requiring antibiotics since study enrollment^1^	11 (0.17)	2 (0.03)	**0.006**
n/a	14	2	
Maternal atopic diseases (self-report)^2^			
Asthma (ever)	27 (0.35)	2 (0.03)	**<0.0001**
n/a	2	3	
Seasonal allergies (current)	52 (0.69)	7 (0.12)	**<0.0001**
n/a	4	4	
Eczema (ever)	24 (0.31)	2 (0.03)	**<0.0001**
n/a	2	3	
Food allergy (ever)	21 (0.28)	7 (0.09)	**0.006**
n/a	4	3	
Paternal atopic diseases (self-report)			
Asthma (current)	10 (0.14)	1 (0.01)	**0.009**
n/a	6	6	
Seasonal allergies (current)	27 (0.67)	13 (0.18)	**<0.0001**
n/a	39	60	
Eczema (current)	9 (0.13)	2 (0.03)	0.06
n/a	7	6	
Food allergy (current)	11 (0.15)	3 (0.04)	**0.05**
n/a	7	6	
Maternal exposures			
Dogs	41 (0.53)	58 (0.74)	**0.008**
Cats	20 (0.26)	24 (0.31)	0.59
Horse	2 (0.03)	62 (0.80)	**<0.0001**
Cow	2 (0.03)	38 (0.49)	**<0.0001**
Pig	1 (0.01)	6 (0.08)	0.12
Poultry	3 (0.04)	43 (0.55)	**<0.0001**
Unpasteurized cow’s milk	0 (0)	54 (0.69)	**<0.0001**
n/a	1	0	
Paternal exposures			
Horse	2 (0.03)	63 (0.81)	**<0.0001**
Cow	2 (0.03)	41 (0.53)	**<0.0001**
Pig	1 (0.01)	5 (0.06)	0.21
Poultry	2 (0.03)	41 (0.53)	**<0.0001**
n/a	1	0	

*Data were derived from the baseline questionnaire completed during pregnancy unless otherwise noted. For all data, besides age and BMI, Fisher’s exact test was used to determine statistical significance. For age and BMI, a Mann–Whitney U test was used. ROC, Rochester. OOM, Old Order Mennonite. N/A, individuals for which data are missing. BMI, body mass index. ^1^Data derived from 6-week survey. ^2^Ever = current diagnosis or childhood diagnosis that has been outgrown. Bold values indicate significant p-values (p < 0.05).*

**TABLE 3:** Home and lifestyle characteristics.

Characteristic	*N* (%) OR MEAN (95% CI)		
	ROC (*n* = 79)	OOM (*n* = 78)	*P*-value
Number of older children	1.3 (1.0, 1.6)	2.5 (2.0, 3.0)	<0.0001
n/a	6	3	
Private water source	3 (0.04)	65 (0.93)	**<0.0001**
n/a	8	8	
Pesticide use			
Inside home	10 (0.16)	29 (0.48)	**0.0002**
n/a	17	17	
Outside home	30 (0.45)	57 (0.84)	**<0.0001**
n/a	12	10	
Anti-bacterial household cleaner use	48 (0.75)	26 (0.46)	**0.001**
n/a	15	21	
Anti-bacterial dishwashing soap use	35 (0.55)	15 (0.32)	**0.02**
n/a	15	30	
Daily use of hand sanitizer	33 (0.43)	4 (0.05)	**<0.0001**
n/a	3	0	
Bleach use	31 (0.44)	26 (0.38)	0.50
n/a	8	9	
Transportation used outside of the home			
Bicycle	7 (0.12)	54 (0.79)	**<0.0001**
n/a	18	10	
Walking	26 (0.39)	44 (0.66)	**0.003**
n/a	12	11	
Horse and buggy	1 (0.02)	59 (0.98)	**<0.0001**
n/a	17	9	
Car/truck/bus/van (job-related)	40 (0.62)	24 (0.37)	**0.005**
n/a	13	12	
Car/truck/bus/van (not job-related)	68 (0.97)	68 (0.99)	1
n/a	8	9	
Food used regularly in the home			
Dairy	63 (0.83)	75 (0.96)	**0.008**
Egg	62 (0.82)	78 (1)	**<0.0001**
Wheat	65 (0.86)	72 (0.92)	0.2064
Soy	33 (0.43)	16 (0.21)	**0.0031**
Peanuts	59 (0.78)	72 (0.92)	**0.0128**
Tree nuts	45 (0.59)	56 (0.72)	0.1270
Fish	43 (0.57)	39 (0.50)	0.4247
Shellfish	24 (0.32)	5 (0.06)	**<0.0001**
Seeds (sesame, sunflower, etc.)	40 (0.53)	49 (0.63)	0.2534
n/a	3	0	

*For all data Fisher’s exact test was used to determine statistical significance. ROC, Rochester. OOM, Old Order Mennonite. N/A, individuals for which data are missing. Bold values indicate significant p-values (p < 0.05).*

### Infant Characteristics and Exposures

According to study questionnaires, there was a higher rate of vaginal, home births in the OOM population compared to ROC, and fewer OOM infants spent any time in the neonatal intensive care unit (NICU) after birth (Table 4). At 6 months of age, OOM infants were more likely to be breastfed, but this difference did not hold at 12 months of age. Similar to OOM mothers and fathers, OOM infants had an increased exposure to farm animals. However, OOM families had less pets living inside the home. ROC infants were more likely to attend daycare, suggesting increased exposure to other children outside of the home. Additionally, the ROC lifestyle was associated with more frequent bathing of infants in comparison to OOM. Interestingly, OOM infants were less likely to be diagnosed with ear infection or reflux in the first year of life. However, self-reported gastrointestinal (vomiting, diarrhea, reflux, and abdominal pain/colic), respiratory (runny nose/cold and cough), or constitutional (fever and irritability) symptoms did not differ between the groups. ROC infants had a higher rate of antibiotic and overall medication usage within the first year of life and were more likely to receive recommended vaccinations, such as diphtheria, tetanus, and whooping cough (DTAP), measles, mumps, and rubella (MMR), hepatitis B, pneumococcus (PCV), poliovirus (IPV), rotavirus, varicella, Haemophilus influenzae type B (Hib), and influenza, on schedule (Table 5).

**TABLE 4:** Infant demographics and exposures in the first year of life.

Characteristic	*N* (%) OR MEAN (95% CI)		
	Rochester (*n* = 79)	OOM (*n* = 78)	*P*-value
Vaginal delivery	50 (0.63)	74 (0.95)	**<0.0001**
n/a	10	2	
Hospital birth	68 (0.86)	16 (0.21)	**<0.0001**
n/a	9	2	
NICU stay	7 (0.11)	1 (0.01)	**0.02**
n/a	14	1	
Caucasian	70 (0.89)	78 (1.0)	**0.0031**
n/a	2	0	
Male	35 (0.44)	49 (0.63)	**0.0252**
n/a	1	0	
Birth weight (lbs)	8.4 (7.8, 9.0)	8.0 (7.8, 8.3)	0.7320
Birth height (in)	20.5 (20.1, 20.9)	20.6 (20.4, 20.8)	0.9273
Breastfeeding			
Any HM at 6 months	63 (0.80)	75 (0.96)	**0.0242**
n/a	5	0	
Exclusive HM 6 months	28 (0.35)	51 (0.65)	**0.0011**
n/a	5	0	
Any HM at 12 months	47 (0.59)	52 (0.67)	0.7302
n/a	6	1	
Exclusive HM 12 months	2 (0.03)	2 (0.03)	1.0000
n/a	3	1	
Animal exposures			
At least one pet lives in house	48 (0.63)	7 (0.09)	**<0.0001**
n/a	8	2	
Dogs	38 (0.48)	65 (0.83)	**<0.0001**
Cats	18 (0.23)	31 (0.40)	**0.0385**
Horse	1 (0.01)	64 (0.82)	**<0.0001**
Cow	1 (0.01)	38 (0.49)	**<0.0001**
Pig	1 (0.01)	14 (0.18)	**0.0006**
Poultry	1 (0.01)	44 (0.56)	**<0.0001**
Smoking exposure in home	3 (0.04)	0 (0)	0.1201
n/a	3	1	
Attends daycare	28 (0.34)	0 (0)	**<0.0001**
n/a	3	0	
Bathe ≤ 2x/week	20 (0.25)	60 (0.77)	**<0.0001**
n/a	11	2	
Infant antibiotics	20 (0.25)	8 (0.10)	**0.0112**
n/a	5	0	
Infant medications	11 (0.15)	1 (0.01)	**0.0020**
n/a	5	1	
Illnesses diagnosed ever			
Ear infection	17 (0.22)	4 (0.05)	**0.0020**
Colic	3 (0.04)	0 (0)	0.1178
Reflux	8 (0.11)	1 (0.01)	**0.0170**
RSV	3 (0.04)	0 (0)	0.1178
n/a	3	0	
Reported GI symptoms			
6 weeks	13 (0.17)	11 (0.14)	0.6610
6 months	14 (0.18)	8 (010)	0.1717
12 months	4 (0.05)	5 (0.06)	1.0000
n/a	3	0	
Reported respiratory symptoms			
6 weeks	9 (0.12)	5 (0.06)	0.2740
6 months	15 (0.20)	7 (0.09)	0.0673
12 months	16 (0.21)	8 (0.10)	0.0774
n/a	3	0	
Reported constitutional symptoms			
6 weeks	7 (0.09)	6 (0.08)	0.7791
6 months	5 (0.07)	2 (0.03)	0.2730
12 months	3 (0.04)	5 (0.06)	0.7194
n/a	3	0	

*Data was derived from 12-month surveys unless otherwise stated. For all data, besides birth weight and birth height, the Fisher’s exact test was used in order to determine statistical significance. For birth weight and birth height, a Mann–Whitney U test was used. ROC, Rochester. OOM, Old Order Mennonite. NICU, neonatal intensive care unit. HM, Human Milk. RSV, respiratory syncytial virus. N/A, individuals for which data are missing. Bold values indicate significant p-values (p < 0.05).*

**TABLE 5:** Infant vaccinations.

Vaccine	*N* (%)		
	Rochester (*n* = 79)	OOM (*n* = 78)	*P*-value
Vaccinated at all	76 (0.96)	20 (0.26)	**<0.0001**
DTAP			**<0.0001**
3 doses on time*	68 (0.87)	6 (0.08)	
Partial (<3 doses)	7 (0.09)	13 (0.17)	
Not vaccinated	3 (0.04)	58 (0.75)	
Hepatitis B			**<0.0001**
≥2 doses on time*	70 (0.90)	10 (0.13)	
Partial (<2 doses)	3 (0.04)	1 (0.01)	
Not vaccinated	5 (0.06)	66 (0.86)	
MMR			**<0.0001**
≥1 dose on time†	57 (0.73)	9 (0.12)	
Not vaccinated	21 (0.27)	68 (0.88)	
PCV			**<0.0001**
3 doses on time*	66 (0.85)	4 (0.05)	
Partial (<3 doses)	5 (0.06)	6 (0.08)	
Not vaccinated	7 (0.09)	67 (0.87)	
IPV			**<0.0001**
≥2 doses on time^†^	66 (0.85)	15 (0.19)	
Partial (<2 doses)	0 (0)	0 (0)	
Not vaccinated	12 (0.15)	62 (0.81)	
Rotavirus (2 or 3 days)			**<0.0001**
2 doses on time*	65 (0.83)	0 (0)	
Partial (<2 doses)	1 (0.01)	0 (0)	
Not vaccinated	12 (0.15)	77 (1.0)	
Varicella			**<0.0001**
1 dose on time†	37 (0.47)	5 (0.06)	
Not vaccinated	41 (0.53)	72 (0.94)	
Hib			**<0.0001**
3 doses on time*	56 (0.72)	1 (0.01)	
Partial	10 (0.13)	8 (0.10)	
Not vaccinated	12 (0.15)	68 (0.88)	
Influenza (≥1 dose on time*)	49 (0.63)	0 (0)	**<0.0001**
n/a	1	1	

For all data Fisher’s exact test was used in order to determine statistical significance. ROC, Rochester. OOM, Old Order Mennonite. N/A, individuals for which data are missing. *On time = by 12 months of age. ^†^On time = by 18 months of age. Bold values indicate significant p-values (p < 0.05).

### Infant Diet

Fruits, vegetables, infant cereal, peanuts, meat, tree nuts, and eggs in baked products were more likely to be introduced later in infancy for OOM infants (Table 6). Similarly, there is a trend for OOM infants to be introduced to scrambled egg (*p* = 0.0553) later in infancy. Furthermore, OOM infants were more likely to be introduced to unpasteurized milk products by 18 months of age compared to ROC infants. Of the OOM infants introduced to unpasteurized cow’s milk by 18 months of age, the average age of introduction was 11.3 months. However, infants were exposed to unpasteurized milk yogurt/pudding even earlier at 7.6 months. In comparison to OOM infants, ROC infants were exposed to a greater variety of foods, such as infant cereal, peanuts, fish, seeds, tree nuts, shellfish, and soy by 18 months of age.

**TABLE 6:** Food introduction.

Food	*N* (%)			Mean months (95% CI)		
	Rochester (*n* = 71)	OOM (*n* = 70)	*P*-value	Rochester (*n* = 71)	OOM (*n* = 70)	*P*-value
Fruits/vegetables	68 (1.0)	67 (0.97)	n/a	6.1 (5.7, 6.4)	7.7 (7.2, 8.3)	**<0.0001**
Infant cereal (rice, oat)	59 (0.87)	39 (0.57)	**0.0001**	6.0 (5.6, 6.3)	8.5 (7.6, 9.4)	**<0.0001**
Peanut	61 (0.90)	39 (0.57)	**<0.0001**	7.8 (7.1, 8.4)	9.8 (9.0, 10.7)	**<0.0001**
Meat	63 (0.93)	60 (0.87)	0.3686	8.0 (7.4, 8.6)	9.8 (9.2, 10.5)	**<0.0001**
Milk yogurt/pudding (store bought)	54 (0.79)	40 (0.58)	**0.0096**	8.2 (7.3, 9.1)	7.7 (6.9, 8.5)	0.9263
Milk yogurt/pudding pasteurized (homemade)	13 (0.19)	15 (0.23)	0.1226	8.6 (6.5, 10.7)	7.0 (6.7, 9.3)	0.9162
Milk yogurt/pudding unpasteurized (homemade)	3 (0.04)	38 (0.55)	**<0.0001**	6.7 (3.8. 9.5)	7.6 (6.9, 8.4)	0.5007
Egg in baked products	56 (0.82)	44 (0.64)	**0.0204**	7.5 (6.9, 8.1)	9.0 (8.2, 9.7)	**0.0008**
Scrambled egg	59 (0.87)	62 (0.90)	0.6056	8.0 (7.4, 8.7)	8.9 (8.2, 9.7)	0.0553
Dairy in baked products	57 (0.84)	43 (0.62)	**0.0067**	8.4 (7.7, 9.0)	9.0 (8.3, 9.7)	0.1255
Kefir (store bought)	3 (0.04)	3 (0.04)	1.0000	6.3 (4.9, 7.8)	6.0 (1.0, 11.0)	>0.9999
Kefir (homemade)	0 (0)	1 (0.01)	1.0000	n/a	11.0	n/a
Cow’s milk pasteurized	41 (0.60)	10 (0.14)	**<0.0001**	11.6 (10.9, 12.3)	11.3 (9.9, 12.7)	0.0753
Cow’s milk unpasteurized	1 (0.01)	42 (0.61)	**<0.0001**	14.0	11.3 (10.4, 12.2)	n/a
Goat’s milk pasteurized	4 (0.06)	1 (0.01)	0.2084	8.5 (3.7, 13.3)	11.0	n/a
Goat’s milk unpasteurized	1 (0.01)	6 (0.09)	0.1154	6.0	9.0 (7.5, 10.5)	n/a
Fish	48 (0.71)	12 (0.17)	**<0.0001**	9.4 (8.6, 10.3)	10.0 (8.4, 11.7)	0.4649
Seeds	38 (0.56)	11 (0.16)	**<0.0001**	10.1 (9.0, 11.2)	11.8 (9.7, 13.9)	0.0999
TREE NUTS	45 (0.66)	9 (0.13)	**<0.0001**	8.4 (7.5, 9.3)	13.2 (11.3, 15.2)	**<0.0001**
SHELLFISH	29 (0.43)	1 (0.01)	**<0.0001**	9.2 (8.1, 10.4)	8.0	n/a
SOY	36 (0.53)	1 (0.01	**<0.0001**	8.4 (7.3, 9.4)	16.0	n/a
N/A	3	1		3	1	

*Fisher’s exact test was used to determine statistical significance of number of infants that had food introduced by 18 months of age. Mann–Whitney U test was used to determine statistical significance of age (months) food was introduced. ROC, Rochester. OOM, Old Order Mennonite. N/A, individuals for which data are missing. Bold values indicate significant p-values (p < 0.05).*

### Infant Allergic Sensitization and Allergic Disease Outcomes

**FIGURE 3:**
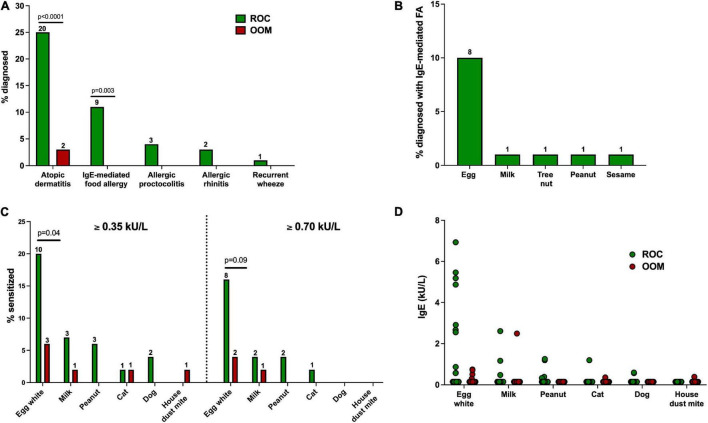
Atopic disease outcomes and sensitization at 12 months of age. Numbers on top of bars in **(A–C)** refer to the absolute number of children represented. **(A)** Proportion of ROC and OOM children with physician-diagnosed allergic diseases. **(B)** Proportion of ROC children diagnosed with IgE-mediated FA. No OOM children were diagnosed with FA. **(C)** Proportion of ROC (*n* = 46–49) and OOM (*n* = 50) children with sensitization to select food and aeroallergens measured by ImmunoCAP using two different cutoffs (≥0.35 kU/L and ≥0.7 kU/L). **(D)** Specific IgE levels in ROC (*n* = 46–49) and OOM (*n* = 50) children. Levels not detectable are displayed as half of the detection limit (0.15 kU/L). For categorical variables, Fisher’s exact test was used to determine statistical significance between OOM and ROC groups. A Mann–Whitney *U* test was used for continuous variables, which all followed a non-normal distribution as assessed by a Shapiro–Wilk test. ROC, Rochester. OOM, Old Order Mennonite.

In Rochester cohort, 20 infants (25%) were diagnosed with AD, 9 infants (11%) with IgE-mediated FA, 3 infants (4%) with allergic proctocolitis, 2 infants (3%) with ARC, and 1 infant (1%) with recurrent wheeze by 12 months of age; in OOM cohort, 2 infants (3%) were diagnosed with AD (Figure 3A). All three infants diagnosed with FA or AP were among the ROC population, 2 of which were diagnosed with both FA and AP (see Table 7 for specifics of the atopic outcomes). Among infants with IgE-mediated FA, 6 were allergic to egg, 1 to egg and peanut, 1 to egg and sesame, and 1 to milk and tree nut (Figure 3B). These data suggest that both groups indeed can be used as a model of either protective or predisposing immune development.

**TABLE 7:** Description of atopic outcomes displayed by 12 months of age.

Group-ID	AD (age of onset)	IgE-mediated FA (age of onset^1^)	Proctocolitis (age of onset^1^)	Food SPT	Food-specific IgE levels (kU/L)	FA symptoms	Allergic rhinitis, wheezing
OOM-24	+ (2–3 months)						
OOM-52	+ (1.5 months)						
ROC-209	+ (9 months)						
ROC-213	+ (11 months)	Milk (11 months) Cashew (24 months)	Milk (1 months)	Milk (+) Cashew (+) Brazil nut (+) Hazelnut (+)	Milk: 1.07	Hives upon milk ingestion; rash with cashew	
ROC-220	+ (3 months)	Egg (15 months)		Egg (+)	Egg: 5.55	Eyelid swelling upon ingestion of baked egg	
ROC-221	+ (9 months)						
ROC-223	+ (5 months)						
ROC-225	+ (10 months)	Peanut (9 months) Egg (9 months)		Peanut (+) Egg (+)	Peanut: 1.2 Egg: (<0.35)	OFC with peanut: nasal congestion, sneezing, cough; OFC with egg: hives and congestion	Allergic rhinitis symptoms with dog; later sensitization with cat and dog.
ROC-229	+ (6 months)						
ROC-240	+ (6 months)						
ROC-241	+ (2 months)						
ROC-242	+ (5 months)						
ROC-246	+ (6 months)						
ROC-256	+ (4 months)						
ROC-261	+ (12 months)	Egg (6 months)		Egg (+)	Egg: 3.07	Hives upon egg ingestion	
ROC-264	+ (12 months)	Egg (12 months) Pistachio sensitization (12 months)		Egg (+) Pistachio (+)	Egg: 2.9	Immediate vomiting upon egg ingestion, never ingested pistachio	
ROC-270	+ (4 months)	Egg (5 months) Sesame (5 months)		Egg (+) Sesame (+)	Egg: 0.58 Sesame: 0.46	Hives upon egg and hummus ingestion	Allergic rhinitis and sensitization to dog
ROC-271		Egg sensitization (10 mo)	Milk (3 months)	Egg (+)	Milk, egg: (<0.35)	Never ingested egg, deferred OFC	Wheezing illness
ROC-280	+ (6 months)	Egg (6 months)		Egg (+)	Egg: 2.55	Delayed vomiting (4 h) repeatedly with scrambled egg	
ROC-281	+ (6 months)						
ROC-284			Beef-derived products, egg, milk, soybean (5 months)				
ROC-286	+ (12 months)	Egg (6 months) Almond sensitization (8 months)		Egg (+) Almond (+)	Egg: 4.87	Hives upon skin contact to scrambled and raw egg (never ingested); almond tolerated at 16 months	
ROC-288	+ (8 months)						
ROC-293	+ (12 months)	Egg (11 months)		Egg (+)	Egg: 5.18	Hives upon egg ingestion	

*^1^Symptoms later confirmed as food allergy via clinical examination and allergy testing. AD, atopic dermatitis; FA, food allergy; SPT, skin prick test; OOM, Old Order Mennonite; ROC, Rochester; OFC, oral food challenge.*

As previously discussed, we are continuing to follow up with these cohorts past 12 months of age and collect data on allergic outcomes on a rolling basis. To date, 74 ROC and 76 OOM subjects are still enrolled in the ongoing study and undergoing follow-up. Our study was powered based on expected rates of 15% FA and 30% of AD. By 12 months of age, the rates of atopic disease in our study are consistent with those anticipated rates with additional diagnoses to be made through out the ongoing follow-up.

At 12 months of age, specific IgE levels to food and aeroallergens were measured by ImmunoCAP. An IgE level ≥0.35 kU/L was used as the cutoff to include low-level sensitization, and 0.7 kU/L was used as a cutoff for moderate-level sensitization (Figures 3C,D). A greater proportion of ROC infants were sensitized to egg white. Additionally, there was an increased proportion of ROC children sensitized to milk and peanut, though the difference did not reach statistical significance. No OOM infants were sensitized to peanut or dog, while no ROC infants appeared sensitized to house dust mites. Not only was sensitization more prevalent among ROC infants, they also appeared to have higher levels of sensitization to allergens compared to sensitized OOM counterparts.

### Multiple Correspondence Analysis

**FIGURE 4:**
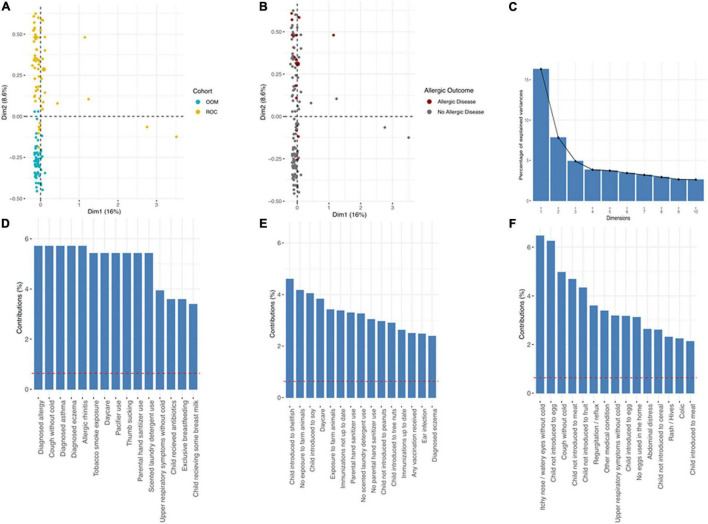
Multiple correspondence analysis (MCA). **(A)** Scatter plot depicting OOM vs. ROC cohort, **(B)** scatter plot depicting physician diagnosis of allergic disease by clinical diagnoses (not parental report in surveys). **(C)** Scree plot depicting % variation explained by each dimension and the 15 variables with largest contribution in dimension **(D)** one, **(E)** two, and **(F)** three.

To investigate the impact of multiple categories in the survey data simultaneously we performed Multiple Correspondence Analysis (MCA). The MCA analysis separated OOM and ROC cohorts (Figure 4A). However, MCA analysis did not separate atopic and non-atopic infants (Figure 4B). Percent variance was primarily explained by 3 dimensions (Figure 4C). Overall ROC cohort has more variation than OOM with few outliers represented by dimension 1 (d1) of MCA. Groups with the d1 was dominated by allergy related observations (Figure 4D). Interestingly, most of the variation between two cohorts was observed across dimension 2 (d2), (Figure 4E). The d2 was dominated by exposure to soy, shellfish and farm animals. Among other variables which had higher weights on d2 were use of sanitizer, daycare and immunization. The third dimension was dominated by exposure to specific foods and upper respiratory symptoms (Figure 4F).

## Discussion

**FIGURE 5:**
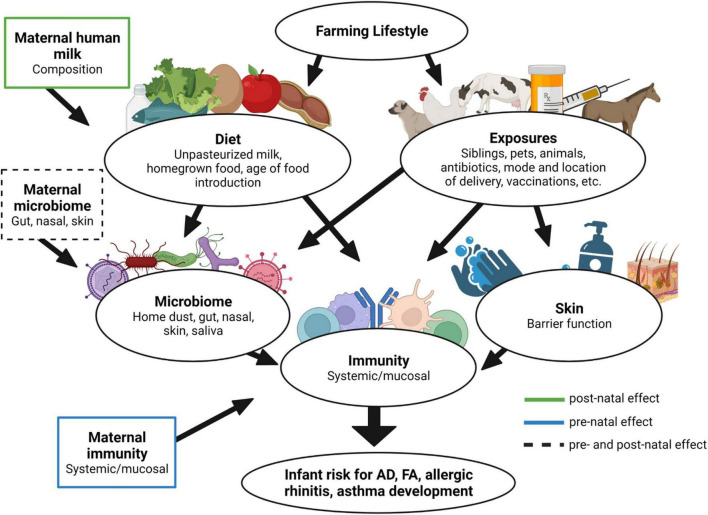
Farm lifestyle impact on development of allergy in early life. Differential diet and microbial exposures due to the farm lifestyle can influence several aspects of infant well-being. Moreover, aspects of the maternal lifestyle can additionally contribute during pre- and postnatal periods, ultimately modulating infant immune function and risk for the development of allergy.

There is clearly a divergence in lifestyle between ROC and OOM impacting several aspects of infant diet and exposures. Furthermore, the early allergic manifestations of FA and AD were robustly present in ROC infants, providing further evidence for utility of OOM and Rochester high-risk populations as model populations for protection or high risk for development of atopic diseases, respectively. Through our vast longitudinal biospecimen collection we will evaluate the biomarkers relating to development of the infant microbiome, systemic and mucosal immunity in both populations. Results from these study can be used as an initial training set for biomarker discoveries. By comparing high risk vs. low-risk populations, the proposed research will investigate for example whether accelerated development of regulatory T cells and IgA responses are biomarkers for “protective” immune development, and whether T cell effector populations are associated with early phases in allergic sensitization. Furthermore, our studies will identify differences in gut microbiome and fecal bacteria inducing IgA responses, and assess the association of specific IgA immune responses with clinical tolerance, i.e., protection from early-onset AD and FA. Additionally, in our study we also have the opportunity to evaluate the maternal influence (breast milk, microbiome, and immunity) on infant immune development and predisposition toward allergic sensitization. Our overall hypothesis is that both maternal and infant lifestyle influence the development of the infant gut, skin and airway microbiome, skin barrier function, and mucosal immunity contributing to the risk of allergy development (Figure 5).

The rate of atopic diseases in ROC infants at 12 months was high but in line with predetermined estimates based on literature in inner city and western populations and family history of atopic disease in this population. Surprisingly, the age of food introduction in OOM infants was higher and the diversity of foods was lower, although their rate of food allergic diseases was lower. This may appear opposite to recent reports on early age of introduction and higher diversity of foods being associated with lower rate of allergies to foods (29–33). However, this finding may indicate that farming/OOM lifestyle is significantly protective against atopic diseases irrespective of other dietary and lifestyle factors. Understanding the biomarkers of such powerful protection can be important for future interventional strategies.

Biomarker discovery requires pre-diagnostic samples from cases that are not available in traditional case–control studies, but can be collected in prospective designs. In biomarker studies utilizing large cohorts, however, the task of understanding individual differences and relationships to the clinical phenotype is only possible with *deep phenotyping* of these clinical correlates, which is resource intensive (34). E.g., immunophenotyping with multiparameter flow cytometry of a large population-based cohort may not be possible due to high cost. An alternative approach is nested case-control setup within a larger cohort study. This pre-selection of the patient population on the basis of their clinical outcome can aid the identification of prognostic factors thereby increasing the power of the study (less samples are used to identify an effect) (35). For example, instead of selecting a random cohort of patients from the patient population, one could select a group of patient who have developed the disease in question (those developing allergy), and a group of patients who have not developed the disease in question (those who have not developed allergy), as extreme sample selection. Our approach here proposes recruitment of high risk and low risk infants at birth as examples of extreme phenotypes as a first step. Such results can provide a “quick and dirty” model for logical checking of hypotheses, but is not appropriate for prognostic modeling (35). To avoid spurious findings, validation and replication studies are required.

The strengths of the study include access to a truly unique agrarian, religious population whose lifestyle represents that of about 100 years ago. Therefore, it provides an unprecedented opportunity to assess the development of infant systemic and mucosal innate and adaptive immunity and early atopic diseases in conditions that mirror those seen prior to the sharp rise in allergic disease. It complements the ongoing Wisconsin Infant Study Cohort (WISC), which is a prospective birth cohort study of infants exposed or not exposed to farm environments with the primary outcome being respiratory viral illness burden in the first 2 years and secondary outcome being allergic sensitization, allergic disease and antiviral innate immune maturation (36). More importantly, we have longitudinal assessment of these infants over time, which can reveal associations between early infant exposures, diet and microbiome and later trajectory of immune function, which can infer causal relationships. Diagnoses of atopic disease have been performed by study physicians during in-person study visits using standard criteria of history, physical exam, skin prick and specific IgE measurement as well as oral food challenges for those in whom major food groups (milk, egg, and peanut) have not been introduced but there is evidence of sensitization.

Limitations include homogenous genetic background of the OOM, especially when contrasted with the ROC diverse high-risk population, with the possibility of an impact of genetic factors on allergy susceptibility. However, because this study was designed to assess the biomarkers of protective immunity and biomarkers of early allergic sensitization process, the many differences in lifestyles or potential differences in genetics of the populations is of less importance. The populations were selected based on likely different rates of atopic disease as examples of extreme populations, in which the initial biomarker discoveries can be made. This study was not designed to assess the factors or mechanisms responsible for or contributing to the differences in the incidence of allergic diseases. Furthermore, the remarkable increase within the last few decades suggests that the lifestyle or microbiome changes are responsible for the rapid increase in allergic disorders, and it is not due to genetics. This is further supported by European lifestyle studies (5, 37, 38) and those in the North America (7, 39) that have identified the farming lifestyle to be protective against allergic diseases and asthma. Assessment of possible genetic differences will be performed in this study using buccal swabs collected to assess susceptibility genes between the populations in order to understand gene-environment interactions. Lastly, the population where the biomarkers are intended to be utilized should also serve as the population where they are being developed. This speaks for the choice of a high-risk population when assessing markers of sensitization as this group would benefit the most from screening for risk of development of allergic diseases in order to trial the efficacy of prevention strategies in the future.

These studies of immune system development in a population protected from and in those at risk for allergic diseases will provide critical new knowledge about the development of the mucosal and systemic immunity and lay the groundwork for future studies of prevention of allergic diseases.

## Collaborative Working Group

Jeanne Lomas^1,2^, Maria H. Slack^1,2^, Puja Sood Rajani^1,2^, Jessica Stern^1,2^, Emily Weis^1,2^, Theresa Bingemann^1,2^, Shahzad S. Mustafa^3^, Allison Ramsey^3^, Barbara Johnson^1^, Kaili Widrick^1^ and Allison Leadley^1^

^1^ Division of Allergy and Immunology, Center for Food Allergy, Department of Pediatrics, University of Rochester School of Medicine and Dentistry, Golisano Children’s Hospital, Rochester, NY, United States

^2^ Division of Allergy, Immunology, and Rheumatology, Department of Medicine, University of Rochester School of Medicine and Dentistry, Rochester, NY, United States

^3^ Allergy, Immunology, and Rheumatology, Rochester Regional Hospital, Rochester, NY, United States

## Ethics Statement

The studies involving human participants were reviewed and approved by University of Rochester Medical Center RSRB. Written informed consent to participate in this study was provided by the participants’ legal guardian/next of kin.

## Author Contributions

KJ, CM, AS, and RL conceived the overall study and supervised the research. KJ, ED, EB, CJ, CP, EC, MK, AS, ND, JM, JT, AS, and the Collaborative Working Group contributed to the literature search, data collection, and data analysis. KJ, ED, EB, CJ, CP, and JT provided the figures and drafted the manuscript, with additional input from all authors. All authors contributed to the article and approved the submitted version.

## Conflict of Interest

KJ has received research funding from Janssen Research & Development, Food Allergy Research and Education and Aimmune. She is a consultant for DBV Technologies and Janssen and received royalties from Up-To-Date and declares no other conflicts of interest. Other authors declare no conflicts of interest.

## Publisher’s Note

All claims expressed in this article are solely those of the authors and do not necessarily represent those of their affiliated organizations, or those of the publisher, the editors and the reviewers. Any product that may be evaluated in this article, or claim that may be made by its manufacturer, is not guaranteed or endorsed by the publisher.

## References

[1] Clemente JC Ursell LK Parfrey LW Knight R. The impact of the gut microbiota on human health: an integrative view. *Cell.* (2012) 148:1258–70. 10.1016/j.cell.2012.01.035 22424233 PMC5050011

[2] Virgin HW. The virome in mammalian physiology and disease. *Cell.* (2014) 157:142–50. 10.1016/j.cell.2014.02.032 24679532 PMC3977141

[3] Tsakok T Marrs T Mohsin M Baron S du Toit G Till S Does atopic dermatitis cause food allergy? A systematic review. *J Allergy Clin Immunol.* (2016) 137:1071–8. 10.1016/j.jaci.2015.10.049 26897122

[4] Pawankar R. Allergic diseases and asthma: a global public health concern and a call to action. *World Allergy Organ J.* (2014) 7:12. 10.1186/1939-4551-7-12 24940476 PMC4045871

[5] von Mutius E Vercelli D. Farm living: effects on childhood asthma and allergy. *Nat Rev Immunol.* (2010) 10:861–8. 21060319 10.1038/nri2871

[6] Holbreich M Genuneit J Weber J Braun-Fahrlander C Waser M von Mutius E. Amish children living in northern Indiana have a very low prevalence of allergic sensitization. *J Allergy Clin Immunol.* (2012) 129:1671–3. 10.1016/j.jaci.2012.03.016 22513133

[7] Stein MM Hrusch CL Gozdz J Igartua C Pivniouk V Murray SE Innate immunity and asthma risk in amish and hutterite farm children. *N Engl J Med.* (2016) 375:411–21. 10.1056/NEJMoa1508749 27518660 PMC5137793

[8] Martina C Looney RJ Marcus C Allen M Stahlhut R. Prevalence of allergic disease in old order mennonites in New York. *Ann Allergy Asthma Immunol.* (2016) 117:562–3.e1. 10.1016/j.anai.2016.08.023 27612936 PMC5708853

[9] Ege MJ Mayer M Normand AC Genuneit J Cookson WO Braun-Fahrlander C Exposure to environmental microorganisms and childhood asthma. *N Engl J Med.* (2011) 364:701–9. 10.1056/NEJMoa1007302 21345099

[10] Lluis A Depner M Gaugler B Saas P Casaca VI Raedler D Increased regulatory T-cell numbers are associated with farm milk exposure and lower atopic sensitization and asthma in childhood. *J Allergy Clin Immunol.* (2014) 133:551–9. 10.1016/j.jaci.2013.06.034 23993223

[11] Schuijs MJ Willart MA Vergote K Gras D Deswarte K Ege MJ Farm dust and endotoxin protect against allergy through A20 induction in lung epithelial cells. *Science.* (2015) 349:1106–10. 10.1126/science.aac6623 26339029

[12] Lundell AC Hesselmar B Nordström I Adlerberth I Wold AE Rudin A. Higher B-cell activating factor levels at birth are positively associated with maternal dairy farm exposure and negatively related to allergy development. *J Allergy Clin Immunol.* (2015) 136:1074–82.e3. 10.1016/j.jaci.2015.03.022 25936566

[13] Ludka-Gaulke T Ghera P Waring SC Keifer M Seroogy C Gern JE Farm exposure in early childhood is associated with a lower risk of severe respiratory illnesses. *J Allergy Clin Immunol.* (2018) 141:454–6.e4. 10.1016/j.jaci.2017.07.032 28870458 PMC5758418

[14] Fujimura KE Sitarik AR Havstad S Lin DL Levan S Fadrosh D Neonatal gut microbiota associates with childhood multisensitized atopy and T cell differentiation. *Nat Med.* (2016) 22:1187–91. 10.1038/nm.4176 27618652 PMC5053876

[15] Abrahamsson TR Jakobsson HE Andersson AF Bjorksten B Engstrand L Jenmalm MC. Low diversity of the gut microbiota in infants with atopic eczema. *J Allergy Clin Immunol.* (2012) 129:434–40.e1-2. 10.1016/j.jaci.2011.10.025 22153774

[16] Azad MB Konya T Guttman DS Field CJ Sears MR HayGlass KT Infant gut microbiota and food sensitization: associations in the first year of life. *Clin Exp Allergy.* (2015) 45:632–43. 10.1111/cea.12487 25599982

[17] Savage JH Lee-Sarwar KA Sordillo J Bunyavanich S Zhou Y O’Connor G A prospective microbiome-wide association study of food sensitization and food allergy in early childhood. *Allergy.* (2018) 73:145–52. 10.1111/all.13232 28632934 PMC5921051

[18] Arrieta MC Stiemsma LT Dimitriu PA Thorson L Russell S Yurist-Doutsch S Early infancy microbial and metabolic alterations affect risk of childhood asthma. *Sci Transl Med.* (2015) 7:307ra152. 10.1126/scitranslmed.aab2271 26424567

[19] Depner M Taft DH Kirjavainen PV Kalanetra KM Karvonen AM Peschel S Maturation of the gut microbiome during the first year of life contributes to the protective farm effect on childhood asthma. *Nat Med.* (2020) 26:1766–75. 10.1038/s41591-020-1095-x 33139948

[20] Seppo AE Bu K Jumabaeva M Thakar J Choudhury RA Yonemitsu C Infant gut microbiome is enriched with *Bifidobacterium longum* ssp. infantis in old order mennonites with traditional farming lifestyle. *Allergy.* (2021) 76:3489–503. 10.1111/all.14877 33905556 PMC9230048

[21] Henrick BM Rodriguez L Lakshmikanth T Pou C Henckel E Arzoomand A Bifidobacteria-mediated immune system imprinting early in life. *Cell.* (2021) 184:3884–98.e11. 10.1016/j.cell.2021.05.030 34143954

[22] Phillips JT Stahlhut RW Looney RJ Jarvinen KM. Food allergy, breastfeeding, and introduction of complementary foods in the New York old order mennonite community. *Ann Allergy Asthma Immunol.* (2020) 124:292–4.e2. 10.1016/j.anai.2019.12.019 31923545

[23] Seppo AE Choudhury R Pizzarello C Palli R Fridy S Rajani PS Traditional farming lifestyle in old older mennonites modulates human milk composition. *Front Immunol.* (2021) 12:741513. 10.3389/fimmu.2021.741513 PMC8545059 34707611

[24] Martina CA Weiss B Swan SH. Lifestyle behaviors associated with exposures to endocrine disruptors. *Neurotoxicology.* (2012) 33:1427–33. 10.1016/j.neuro.2012.05.016 22739065 PMC3641683

[25] Zimet GD Dahlem NW Zimet SG Farley GK. The multidimensional scale of perceived social support. *J Pers Assess.* (1988) 52:30–41. 10.1080/00223891.1990.9674095 2280326

[26] Boyce JA Assa’ad A Burks AW Jones SM Sampson HA Wood RA Guidelines for the diagnosis and management of food allergy in the united states: summary of the niaid-sponsored expert panel report. *J Allergy Clin Immunol.* (2010) 126:1105–18. 21134568 10.1016/j.jaci.2010.10.008 PMC4241958

[27] McGowan EC Bloomberg GR Gergen PJ Visness CM Jaffee KF Sandel M Influence of early-life exposures on food sensitization and food allergy in an inner-city birth cohort. *J Allergy Clin Immunol.* (2015) 135:171–8. 10.1016/j.jaci.2014.06.033 25129677 PMC4440482

[28] Wood RA Bloomberg GR Kattan M Conroy K Sandel MT Dresen A Relationships among environmental exposures, cord blood cytokine responses, allergy, and wheeze at 1 year of age in an inner-city birth cohort (Urban Environment and Childhood Asthma study). *J Allergy Clin Immunol.* (2011) 127:913–9.e1-6. 10.1016/j.jaci.2010.12.1122 21333343 PMC3070829

[29] D’Auria E Peroni DG Sartorio MUA Verduci E Zuccotti GV Venter C. The role of diet diversity and diet indices on allergy outcomes. *Front Pediatr.* (2020) 8:545. 10.3389/fped.2020.00545 PMC7522364 33042906

[30] Venter C Greenhawt M Meyer RW Agostoni C Reese I du Toit G EAACI position paper on diet diversity in pregnancy, infancy and childhood: novel concepts and implications for studies in allergy and asthma. *Allergy.* (2020) 75:497–523. 10.1111/all.14051 31520486

[31] Du Toit G Roberts G Sayre PH Bahnson HT Radulovic S Santos AF Randomized trial of peanut consumption in infants at risk for peanut allergy. *N Engl J Med.* (2015) 372:803–13. 25705822 10.1056/NEJMoa1414850 PMC4416404

[32] Venter C Maslin K Holloway JW Silveira LJ Fleischer DM Dean T Different measures of diet diversity during infancy and the association with childhood food allergy in a UK birth cohort study. *J Allergy Clin Immunol Pract.* (2020) 8:2017–26. 10.1016/j.jaip.2020.01.029 32004745

[33] Perkin MR Logan K Marrs T Radulovic S Craven J Flohr C Enquiring About Tolerance (EAT) study: feasibility of an early allergenic food introduction regimen. *J Allergy Clin Immunol.* (2016) 137:1477–86.e8. 10.1016/j.jaci.2015.12.1322 26896232 PMC4852987

[34] McPartland JC Bernier RA Jeste SS Dawson G Nelson CA Chawarska K The autism biomarkers consortium for clinical trials (ABC-CT): scientific context, study design, and progress toward biomarker qualification. *Front Integr Neurosci.* (2020) 14:16. 10.3389/fnint.2020.00016 PMC7173348 32346363

[35] Hall JA Brown R Paul J. An exploration into study design for biomarker identification: issues and recommendations. *Cancer Genomics Proteomics.* (2007) 4:111–9. 17878515

[36] Seroogy CM VanWormer JJ Olson BF Evans MD Johnson T Cole D Respiratory health, allergies, and the farm environment: design, methods and enrollment in the observational Wisconsin Infant Study Cohort (WISC): a research proposal. *BMC Res Notes.* (2019) 12:423. 10.1186/s13104-019-4448-0 PMC6636141 31311588

[37] Riedler J Braun-Fahrlander C Eder W Schreuer M Waser M Maisch S Exposure to farming in early life and development of asthma and allergy: a cross-sectional survey. *Lancet.* (2001) 358:1129–33. 10.1016/S0140-6736(01)06252-3 11597666

[38] Loss G Apprich S Waser M Kneifel W Genuneit J Büchele G The protective effect of farm milk consumption on childhood asthma and atopy: the GABRIELA study. *J Allergy Clin Immunol.* (2011) 128:766–73.e4. 10.1016/j.jaci.2011.07.048 21875744

[39] Tantoco JC Elliott Bontrager J Zhao Q DeLine J Seroogy CM. The Amish have decreased asthma and allergic diseases compared with old order Mennonites. *Ann Allergy Asthma Immunol.* (2018) 121:252–3.e1. 10.1016/j.anai.2018.05.016 29802980 PMC7744242

