# Supplementary material for: Biomarkers of Development of Immunity and Allergic Diseases in Farming and Non-farming Lifestyle Infants: Design, Methods and 1 Year Outcomes in the “Zooming in to Old Order Mennonites” Birth Cohort Study
Source: Front Pediatr. 2022 Jul 6;10:916184. doi: 10.3389/fped.2022.916184 (PMC9299374; doi:10.3389/fped.2022.916184)
Supplement: Supplementary file 1 [file Table_1.DOCX]

Supplementary Material

| Table 1. Questionnaire domains and administration schedule | | | | |
| --- | --- | --- | --- | --- |
| Characteristic | **Prenatal** | **6 wks** | **4-6, 12, 18, 24 mo** | **3, 4, 5 yr** |
| Maternal |  |  |  |  |
| Age | **x** |  |  |  |
| Height/weight | **x** | **x** | **x** | **x** |
| Antibiotics/medications | **x** | **x** | **x** | **x** |
| Medications | **x** | **x** | **x** |  |
| Vaccinations (recent) | **x** | **x** |  |  |
| General health | **x** | **x** |  |  |
| Chronic medical conditions | **x** | **x** |  |  |
| Atopic disease history | **x** |  |  |  |
| Vitamins/supplements | **x** |  |  |  |
| Diet | **x** |  |  |  |
| Pet exposure | **x** |  |  |  |
| Farm animal exposure | **x** |  |  |  |
| Perceived social support | **x** |  |  |  |
| Paternal |  |  |  |  |
| Age | **x** |  |  |  |
| Atopic disease history | **x** |  |  |  |
| Farm animal exposure | **x** |  |  |  |
| Home and lifestyle |  |  |  |  |
| House time period | **x** |  |  |  |
| Home construction materials | **x** |  |  |  |
| Home renovations | **x** |  |  |  |
| Heating sources | **x** |  |  |  |
| Water source | **x** |  |  |  |
| Food types eaten by family | **x** |  |  |  |
| Food storage | **x** |  |  |  |
| Transportation | **x** |  |  |  |
| Occupations | **x** |  |  |  |
| Household cleaners | **x** |  |  |  |
| Pesticides | **x** |  |  |  |
| Hair products | **x** |  |  |  |
| Personal care products | **x** |  |  |  |
| Infant/Child |  |  |  |  |
| Mode and location of delivery |  | **x** |  |  |
| Height/weight |  | **x** | **x** | **x** |
| Antibiotics/medications |  | **x** | **x** | **x** |
| Vitamins/supplements |  | **x** | **x** | **x** |
| Vaccinations |  | **x** | **x** | **x** |
| Illnesses, sick visits |  | **x** | **x** | **x** |
| Allergy symptoms |  | **x** | **x** | **x** |
| Household pet exposure |  | **x** | **x** | **x** |
| Farm animal exposure |  | **x** | **x** | **x** |
| Smoke exposure |  | **x** | **x** | **x** |
| Home food exposure |  | **x** | **x** | **x** |
| Childcare |  | **x** | **x** | **x** |
| Bathing practices |  | **x** | **x** | **x** |
| Personal care products |  | **x** | **x** | **x** |
| Breastfeeding history |  | **x** | **x** |  |
| Infant formula use |  | **x** | **x** |  |
| Food introduction/diet history |  |  | **x** | **x** |

| **Table 2. Sample collection inventory** | | | | | | | | |
| --- | --- | --- | --- | --- | --- | --- | --- | --- |
|  | **Prenatal** | **Birth** | **1 wk** | **6 wk** | **6 mo** | **12 mo** | **18 mo** | **24 mo*** |
| ***Mother*** |  |  |  |  |  |  |  |  |
| Blood & derivatives | 58/69 |  |  | 2/5 |  |  |  |  |
| Serum | 77/87 |  |  | 17/11 |  |  |  |  |
| Breast milk |  | 60/52 | 53/55 | 66/66 | 75/61 |  |  |  |
| Stool | 56/59 |  |  | 17/15 |  |  |  |  |
| Urine | 80/93 |  |  |  |  |  |  |  |
| Saliva | 58/76 |  |  | 17/18 |  |  |  |  |
| Skin swab | 57/75 |  |  | 17/18 |  |  |  |  |
| Nasal brush | 53/68 |  |  |  |  |  |  |  |
| Nasal swab | 82/93 |  |  |  |  |  |  |  |
| Placenta |  | 27/33 |  |  |  |  |  |  |
| ***Infant*** |  |  |  |  |  |  |  |  |
| Cord blood |  | 60/46 |  |  |  |  |  |  |
| Peripheral blood |  |  |  |  | 62/54 | 58/53 |  | 60/39 |
| Stool |  |  | 56/52 | 70/68 | 79/73 | 66/72 | 70/51 | 66/51 |
| Saliva |  |  | 49/55 | 70/75 | 77/81 | 77/76 | 73/57 | 73/52 |
| Skin swab |  |  | 48/53 | 64/77 | 77/81 | 78/74 |  | 74/54 |
| Buccal swab |  |  |  | 68/72 |  |  |  |  |
| Nasal brush |  |  |  | 62/76 | 77/80 | 74/73 | 73/56 | 73/50 |
| Nasal swab |  |  |  | 67/76 | 77/82 | 77/74 | 73/56 | 73/53 |
| Nasal wash |  |  |  | 58/71 | 77/80 | 76/72 | 71/54 | 73/54 |
| ***Family*** |  |  |  |  |  |  |  |  |
| House dust |  |  |  | 22/8 | 9/20 | 8/21 | 2/12 | 4/5 |
| Number of samples are shown for OOM and ROC arms of the study separately (OOM/ROC). Numbers include samples collected from all enrolled study subjects, including those that withdrew before 12 months of age. | | | | | | | | |
